# Inhibitory Effects of Oxytocin on Jejunal Migrating Myoelectric Complex Activity in Fasted Rats: Role of Oxytocin and GLP-1 Receptors

**DOI:** 10.3390/life16061029

**Published:** 2026-06-19

**Authors:** Hakan Balcı, Özge Darakcı Saltık, Burcu Hatipoğlu Aktemur, Rümeysa Abdullahoğlu, Ayhan Bozkurt

**Affiliations:** 1 Department of Medical Pharmacology, Faculty of Medicine, Bilecik Şeyh Edebali University, Bilecik 11100, Türkiye; hakan.balci@bilecik.edu.tr; 2 Department of Physiology, Faculty of Medicine, Bilecik Şeyh Edebali University, Bilecik 11100, Türkiye; ozge.saltik@bilecik.edu.tr; 3 Department of Physiology, Faculty of Medicine, Sağlık Bilimleri University, Istanbul 34668, Türkiye; burcu.hatipogluaktemur@sbu.edu.tr; 4 Department of Physiology, Faculty of Medicine, Ondokuz Mayıs University, Samsun 55270, Türkiye; rumeysa.abdullahoglu@omu.edu.tr

**Keywords:** GLP-1 receptor, migrating myoelectric complex, oxytocin, oxytocin receptor, intestinal motility

## Abstract

The migrating myoelectric complex (MMC) is the electrical basis of fasting small intestinal motility. Although oxytocin (OT) regulates gastrointestinal functions through oxytocin receptors (OTRs), its effect on jejunal MMC activity during fasting remains unclear. This study investigated the effects of OT on jejunal MMC activity in fasted rats and evaluated the involvement of OTRs, glucagon-like peptide-1 receptors (GLP-1Rs), and nitric oxide (NO) pathways. Bipolar electrodes were implanted at three jejunal sites in adult male Sprague Dawley rats for MMC recordings. After recovery and 18 h fasting, OT was administered intraperitoneally (4–32 µg/kg) following one hour of basal recording. To assess mechanisms, rats were pretreated with the OTR antagonist atosiban (2 mg/kg), the GLP-1R antagonist exendin (9–39) (200 µg/kg), or the nitric oxide synthase inhibitor NG-nitro-L-arginine (L-NNA; 5 mg/kg) before OT (16 µg/kg). Oxytocin dose-dependently reduced spike frequency and MMC cycle number (*p* < 0.05–0.001 vs. vehicle). Atosiban completely reversed these effects (*p* < 0.001 vs. OT), while exendin (9–39) partially attenuated them (*p* < 0.01–0.001 vs. OT). L-NNA showed no significant effect. These findings indicate that OT inhibits jejunal MMC activity via OTR-dependent mechanisms with partial involvement of GLP-1R signaling but not NO pathways.

## 1. Introduction

The motility of the small intestine is essential for the physiological function of the digestive system. During the fasting period, this motility is generated by the migrating myoelectric complex (MMC), which exhibits a regular, rhythmic pattern consisting of three phases [phase I (quiescence); phase II (irregular electrical activity); phase III (high-frequency, regular spike potentials)] [1]. This complex serves as a critical physiological mechanism for cleansing the intestinal lumen and maintaining control of the intestinal microbial load [2].

Given its important physiological functions, disruption of MMC activity has been associated with several gastrointestinal disorders, including small intestinal bacterial overgrowth, gastroparesis, intestinal pseudo-obstruction, irritable bowel syndrome, and obesity. These observations suggest that MMC plays an important role not only in maintaining normal intestinal physiology but also in the pathophysiology of gastrointestinal motility disorders [2,3,4].

Several neurohumoral mediators have been shown to play important roles in the regulation of MMC activity. Motilin and serotonin have been identified as key stimulatory mediators in the initiation and progression of MMC [5,6,7]. In contrast, several neurohumoral factors, including glucagon-like peptide-1 (GLP-1) and nitric oxide (NO), have been shown to exert tonic inhibitory effects on this process [8,9,10]. However, the neurohumoral control mechanisms of MMC are still not fully understood. Therefore, identifying novel mediators involved in MMC modulation will contribute to a better understanding of the physiological and pathophysiological aspects of gastrointestinal motility [11,12,13].

Oxytocin (OT) is a hypothalamic neuropeptide synthesized primarily in the paraventricular and supraoptic nuclei and is also expressed in peripheral tissues, where it exerts its effects through activation of the Gq protein-coupled oxytocin receptor (OTR). In addition to its well-established roles in uterine contractions and lactation, OT has recently been shown to exert effects on the gastrointestinal system. Evidence suggests that OT and its receptors are present in several regions of the gastrointestinal tract and may influence gastrointestinal motility [11,12,14]. For instance, while some studies have reported that OT inhibits gastric emptying and intestinal transit [11,12,13], other studies have demonstrated that OT exerts stimulatory effects on gastrointestinal motility [12,15]. Although the effects of OT on gastrointestinal motility have been investigated in various studies, no study to date has specifically evaluated its effects on the MMC during the fasting period.

Therefore, the present study aimed to investigate the potential effects of OT on small intestinal MMC activity in fasted rats. In addition, this study aimed to evaluate the involvement of OTRs, GLP-1 receptors (GLP-1Rs), and the nitric oxide (NO) synthesis pathway in mediating these effects using pharmacological inhibition.

## 2. Materials and Methods

### 2.1. The Ethics Statement

The protocols followed the principles presented in the Guide for the Care and Use of Laboratory Animals (NIH Publication No. 865–23), with a focus on minimizing animal distress and ensuring ethical treatment. All procedures were approved by the Animal Studies Ethics Committee of the Faculty of Medicine, Ondokuz Mayıs University, Samsun, Turkey (Approval No: HADYEK 2022/25).

### 2.2. Animals

A total of 73 adult male Sprague Dawley rats, aged 8–10 weeks and weighing 250–300 g, were used in this study. Male rats were selected to avoid potential variability in gastrointestinal motility associated with hormonal fluctuations during the estrous cycle. All animals were allowed to acclimatize to the housing conditions for one week before the experiments. Animals were housed under controlled environmental conditions (22 ± 2 °C, 50–60% relative humidity) with a 12 h light/dark cycle. Food and water were provided ad libitum. Prior to surgery, rats were housed in groups of four per cage. Following surgical implantation of the electrodes, animals were housed individually during the recovery and recording periods.

### 2.3. Experimental Protocols

#### 2.3.1. Electromyography Recordings

Animals were anesthetized using a combination of ketamine (100 mg/kg) and chlorpromazine (0.75 mg/kg), both administered via the intraperitoneal (i.p.) route. A midline abdominal incision was performed to implant 80 µm Ni/Cr wire electrodes at three locations along the small intestine: 10 cm (J1), 20 cm (J2), and 30 cm (J3) distal to the pylorus, enabling electromyographic (EMG) signal recording [16,17,18]. Electrodes were tunneled subcutaneously and securely fixed with dental acrylic to ensure long-term stability during recordings. Postoperative infection was prevented by administering cefazolin (100 mg/kg/day, intramuscularly) for three consecutive days. Following surgery, animals were allowed a 7-day recovery period before the initiation of EMG recordings. During this recovery period, starting on the second postoperative day, rats were habituated to Bollman cages for 2 h daily for four consecutive days to minimize stress and facilitate adaptation to the recording conditions. EMG recordings were initiated on postoperative day 8. To standardize fasting motility patterns, animals were fasted for 18 h before the first recording session while maintaining free access to water. EMG recordings were obtained from conscious rats using a bioamplifier (ML132, ADInstruments, Sydney, Australia) connected to a PowerLab data acquisition system (ML870/P, ADInstruments, Sydney, Australia) [16,17,18,19].

#### 2.3.2. Experimental Design and Groups

The experiment began with a one-hour control period during which three MMC cycles were recorded at all electrode sites (J1, J2, and J3), serving as the baseline for the evaluation of drug effects. EMG recordings continued for at least one hour following drug administration to monitor post-treatment motility. In groups pretreated with antagonists, the antagonists were administered ten minutes prior to OT to ensure effective receptor blockade.

In the control group (vehicle), physiological saline [0.9% NaCl; 1 mL/kg, i.p.]—the solvent used for all experimental compounds—was administered. All treatment groups were compared to this control.

In the first experimental group, designed to evaluate the dose-dependent effects of OT, OT was administered i.p. at doses of 4, 8, 16, and 32 µg/kg, yielding four separate dose–response groups. These doses were initially based on previous studies reporting effective doses in gastrointestinal models ranging from 100 to 240 µg/kg [11,20,21]. However, in our preliminary experiments, significant effects were observed even at much lower doses. Therefore, the dose range of 4–32 µg/kg was selected for the present study.

In the second experimental group, the involvement of OTRs in OT-induced inhibition of MMC activity was evaluated by administering the selective OTR antagonist atosiban (2 mg/kg, i.p.) 10 min before OT (16 µg/kg, i.p.) [22].

In the third experimental group, the nitric oxide synthase inhibitor NG-nitro-L-arginine (L-NNA; 5 mg/kg, i.p.) was given 10 min before OT (16 µg/kg, i.p.) to assess the contribution of nitric oxide signaling to OT-induced inhibition of MMC activity [23].

In the fourth group, the GLP-1R antagonist exendin (9–39) (200 µg/kg, i.p.) was administered 10 min before OT (16 µg/kg, i.p.) to evaluate the involvement of GLP-1 receptors in mediating the effects of OT on MMC patterns [16].

**Figure 1:**
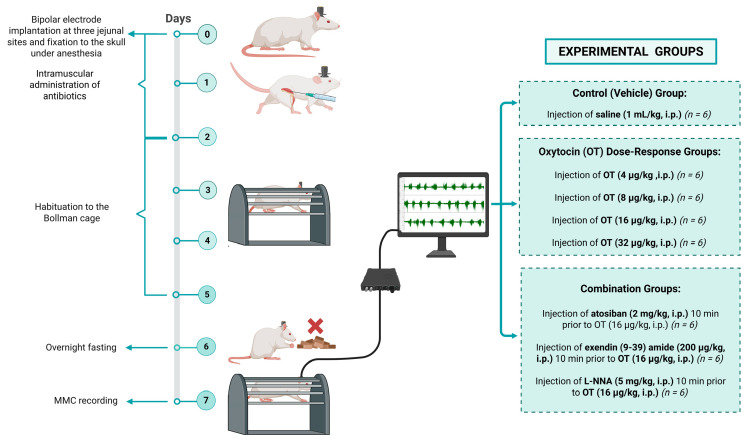
Bipolar electrodes were implanted in the jejunum, followed by recovery, antibiotic treatment, cage habituation, and overnight fasting. A one-hour baseline EMG recording was then performed, followed by i.p. drug administration. Treatment groups included vehicle, OT (4–32 µg/kg), and OT combined with specific antagonists.

The experimental design and treatment groups are illustrated in Figure 1.

In additional control experiments, atosiban (2 mg/kg, i.p.), L-NNA (5 mg/kg, i.p.), and exendin (9–39) (200 µg/kg, i.p.) were also administered alone to evaluate their effects on basal MMC activity. Antagonist-alone control groups were included in the experimental protocol but are not shown in Figure 1 for clarity.

Overall, the study consisted of 11 experimental groups, with six animals per group (*n* = 6). The selected doses of atosiban, exendin (9–39), and L-NNA were chosen based on previous in vivo studies demonstrating effective OTR blockade, GLP-1R blockade, and inhibition of nitric oxide synthase activity, respectively [16,22,23].

### 2.4. Drugs and Chemicals

Oxytocin (Sigma-Aldrich, St. Louis, MO, USA), atosiban (Sigma-Aldrich, St. Louis, MO, USA), N^G^-nitro-L-arginine (L-NNA; Sigma-Aldrich, St. Louis, MO, USA), and exendin (9–39) (Tocris Bioscience, Bristol, UK) were used in the experiments. All compounds were dissolved in sterile physiological saline (0.9% NaCl) immediately before administration. The drugs were administered i.p. at a constant volume of 1 mL/kg body weight.

### 2.5. Data Analysis

Typical interdigestive myoelectric activity of the small intestine is defined by a distinct phase III—the activity front—characterized by intense, high-amplitude spike bursts propagating aborally, followed sequentially by phases I and II. This cyclic pattern repeats rhythmically throughout the fasting state [2]. Prolonged periods (>30 min) exhibiting irregular spiking activity without discernible cyclic organization were classified as disrupted MMC activity. Conversely, intervals devoid of detectable spike potentials were identified as quiescent periods characterized by inhibition of MMC activity. Quantitative analysis of MMC parameters, including spike frequency and the total number of complete MMC cycles, was performed over a one-hour recording period following injections at the J1, J2, and J3 intestinal recording [19]. EMG signals were analyzed offline using LabChart software version 7.3.8 (ADInstruments Pty Ltd., Sydney, Australia).

### 2.6. Statistical Analysis

Sample size estimation was performed using G*Power 3.1 software based on a one-way ANOVA design. The power analysis was performed using a significance level of α = 0.05, a statistical power of 1 − β = 0.85, and an effect size of f = 0.60 according to Cohen’s criteria [24]. The analysis indicated that at least six animals per experimental condition (*n* = 6) were required to detect statistically significant differences among groups. Considering a potential data loss of approximately 10%, a total of 73 animals were planned to be included in the study. Data are presented as mean ± standard error of the mean (SEM), unless otherwise specified. Normality of the data distribution was assessed using the Shapiro–Wilk test. Comparisons among multiple groups were performed using one-way analysis of variance (ANOVA), followed by the Tukey–Kramer post hoc test. The animal was considered the experimental unit for all statistical analyses. MMC parameters obtained from the J1, J2, and J3 recording sites were analyzed separately, with each site including data from six animals per experimental group (*n* = 6). Statistical analyses were performed independently for each recording site [19]. All statistical analyses and graphical representations were carried out using GraphPad Prism (version 8.0.2; GraphPad Software Inc., San Diego, CA, USA), and *p* < 0.05 was considered statistically significant.

## 3. Results

### 3.1. Effect of Oxytocin on MMC

**Figure 2:**
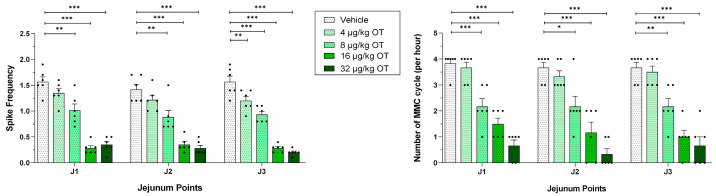
Effect of OT on jejunal MMC activity. Oxytocin caused a dose-dependent reduction in spike frequency (**left**) and the number of MMC cycles (**right**) across jejunal segments (J1–J3). Values are expressed as mean ± SEM (*n* = 6 per group; * *p* < 0.05, ** *p* < 0.01, and *** *p* < 0.001 vs. vehicle).

Spike frequency: I.p. administration of OT produced a clear dose-dependent reduction in spike frequency across all jejunal recording sites. At the lowest dose (4 µg/kg), OT did not significantly alter spike frequency at J1 and J2 compared with vehicle-treated animals (J1: 1.35 ± 0.1 vs. 1.5 ± 0.1; J2: 1.2 ± 0.2 vs. 1.5 ± 0.1; *p* > 0.05). However, a significant reduction was observed at J3 (1.2 ± 0.1 vs. 1.5 ± 0.1; *p* < 0.01). At 8 µg/kg, spike frequency was significantly reduced at all jejunal recording sites (J1: 1.0 ± 0.1; J2: 0.9 ± 0.1; J3: 0.9 ± 0.1; *p* < 0.01–0.001). Higher doses produced a more pronounced suppression of spike activity. At 16 µg/kg, spike frequency decreased to 0.28 ± 0.05 at J1, 0.35 ± 0.06 at J2, and 0.28 ± 0.03 at J3 (*p* < 0.001 for all). Similarly, administration of 32 µg/kg OT resulted in near-complete inhibition of spike activity at all recording sites (J1: 0.35 ± 0.06; J2: 0.28 ± 0.05; J3: 0.20 ± 0.03; *p* < 0.001), consistent with a dose-dependent suppression of spike frequency (Figure 2, left panel).

Number of MMC Cycles: At the 4 µg/kg dose, OT did not significantly affect the number of MMC cycles per hour at any jejunal recording site. Mean values remained comparable to vehicle controls (J1: 3.7 ± 0.2 vs. 3.8 ± 0.2; J2: 3.3 ± 0.2 vs. 3.7 ± 0.2; J3: 3.5 ± 0.2 vs. 3.7 ± 0.2; *p* > 0.05). In contrast, administration of 8 µg/kg OT significantly reduced MMC cycle frequency at all jejunal segments (J1: 2.2 ± 0.3; J2: 2.2 ± 0.4; J3: 2.2 ± 0.3; *p* < 0.05–0.001). At 16 µg/kg, a marked inhibitory effect was observed, with MMC cycle numbers decreasing to 1.5 ± 0.2 at J1, 1.2 ± 0.4 at J2, and 1.0 ± 0.3 at J3 (*p* < 0.001 for all). At the highest dose (32 µg/kg), OT almost completely abolished MMC activity across all jejunal segments, with cycle numbers declining to 0.67 ± 0.21 at J1, 0.33 ± 0.21 at J2, and 0.67 ± 0.33 at J3 (*p* < 0.001 for all), indicating near-complete suppression of fasting jejunal motility (Figure 2, right panel).

Notably, no statistically significant difference was observed between the 16 and 32 μg/kg groups in either spike frequency or the number of MMC cycles (*p* > 0.05), suggesting that no additional inhibitory effect was detected at 32 μg/kg under the present experimental conditions. Consequently, the 16 μg/kg dose was chosen for the combination groups (Figure 2).

### 3.2. The Role of Receptor- and NO-Dependent Pathways in Oxytocin-Induced Modulation of MMC Activity

#### 3.2.1. Effect of Oxytocin Receptor Blockade by Atosiban

**Figure 3:**
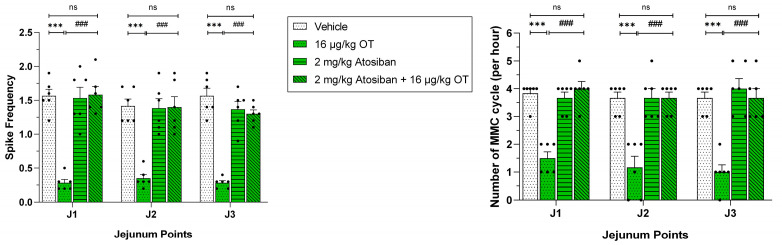
Effect: Pretreatment with atosiban (2 mg/kg, i.p.) completely reversed the inhibitory effect of OT (16 µg/kg) on both spike frequency (**left**) and the number of MMC cycles (**right**) across jejunal segments (J1–J3). Values are expressed as mean ± SEM (*n* = 6 per group; *** *p* < 0.001 vs. vehicle; ^###^ *p* < 0.001 vs. OT; ns (not significant) *p* > 0.05).

Pretreatment with atosiban (2 mg/kg, i.p.) significantly reversed the inhibitory effect of OT on spike frequency, resulting in values comparable to those of the vehicle-treated control group (*p* < 0.001 vs. OT; *p* > 0.05 vs. vehicle) (Figure 3, left panel). Similarly, atosiban pretreatment prevented the OT-induced reduction in the number of MMC cycles, restoring MMC activity to control levels (*p* < 0.001 vs. OT; *p* > 0.05 vs. vehicle) (Figure 3, right panel). Administration of atosiban alone did not significantly affect spike frequency or MMC cycle number compared with vehicle-treated controls (*p* > 0.05) (Figure 3).

#### 3.2.2. Effect of GLP-1 Receptor Blockade by Exendin (9–39)

**Figure 4:**
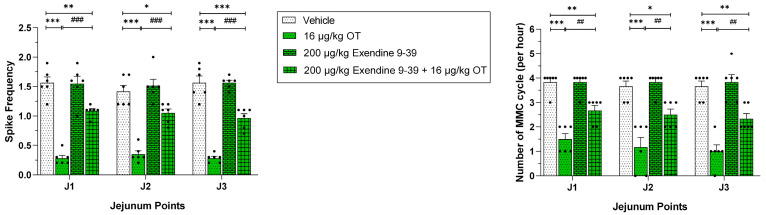
Pretreatment with the GLP-1 receptor antagonist exendin (9–39) (200 µg/kg, i.p.) partially reversed the inhibitory effect of OT (16 µg/kg) on both spike frequency (**left**) and the number of MMC cycles (**right**) across jejunal segments (J1–J3). Values are mean ± SEM (*n* = 6 per group; * *p* < 0.05, ** *p* < 0.01, *** *p* < 0.001 vs. vehicle; ^##^ *p* < 0.01, ^###^ *p* < 0.001 vs. OT).

Pretreatment with the GLP-1 receptor antagonist exendin (9–39) (200 µg/kg, i.p.) partially reversed the inhibitory effects of OT on MMC. Exendin (9–39) pretreatment attenuated the OT-induced inhibition in spike frequency and the number of MMC cycles at J1–J3 (*p* < 0.05–0.001 vs. OT), bringing the values closer to those of the vehicle group but remaining significantly lower than control levels (*p* < 0.05–0.001 vs. vehicle). No significant alterations were observed in the exendin (9–39) group compared with the vehicle group (*p* > 0.05) (Figure 4).

#### 3.2.3. Effect of Nitric Oxide Synthase Inhibition by L-NNA

**Figure 5:**
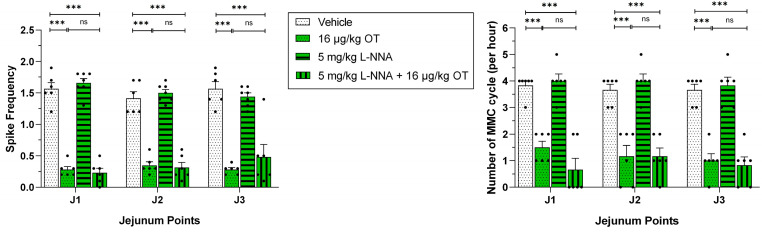
L-NNA pretreatment (5 mg/kg, i.p.) did not alter the OT-induced inhibition of spike frequency (**left**) or the number of MMC cycles (**right**) in jejunal segments (J1–J3). Values are mean ± SEM (*n* = 6 per group; *** *p* < 0.001 vs. vehicle; ns *p* > 0.05).

The inhibitory effect of OT on MMC was not altered by pretreatment with the NOS inhibitor L-NNA (5 mg/kg, i.p.). Pretreatment with L-NNA did not affect the OT-induced decreases in spike frequency and the number of MMC cycles at J1–J3 (*p* > 0.05 vs. OT; *p* < 0.001 vs. vehicle). L-NNA alone had no significant effect compared with the vehicle group (*p* > 0.05) (Figure 5).

**Figure 6:**
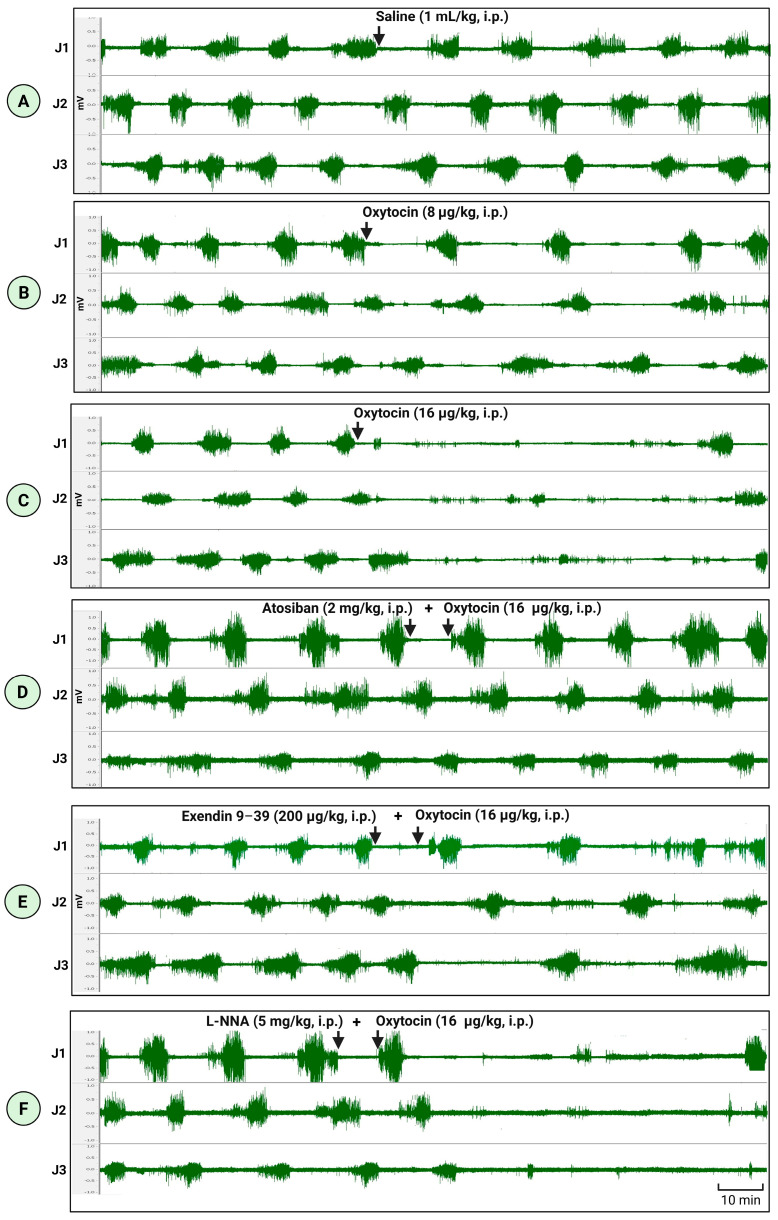
Representative recordings of jejunal MMC activity following OT administration and pretreatment with pharmacological antagonists. (**A**) Saline (1 mL/kg, i.p.); (**B**) OT (8 µg/kg, i.p.); (**C**) OT (16 µg/kg, i.p.); (**D**) atosiban (2 mg/kg, i.p.) + OT (16 µg/kg, i.p.); (**E**) exendin (9–39) (200 µg/kg, i.p.) + OT (16 µg/kg, i.p.); (**F**) L-NNA (5 mg/kg, i.p.) + OT (16 µg/kg, i.p.). Arrows indicate the time of drug administration.

Representative MMC tracings obtained from the vehicle-, OT (8 and 16 µg/kg)-, atosiban + OT-, exendin (9–39) + OT-, and L-NNA + OT-treated groups are shown in Figure 6.

## 4. Discussion

In the present study, we demonstrated that OT exerts a clear dose-dependent inhibitory effect on jejunal MMC activity in fasted rats. The suppression of MMC was characterized by a marked reduction in both spike frequency and the number of MMC cycles. Pretreatment with the selective OTR antagonist atosiban completely reversed this effect. By comparison, the GLP-1R antagonist exendin (9–39) only partially attenuated the inhibitory effect of OT, whereas inhibition of NOS had no effect.

Numerous in vivo studies have demonstrated that OT exerts a suppressive effect on gastrointestinal motility. For instance, intravenous (i.v.) administration of OT at doses of 1–4 µg/kg has been reported to completely abolish peristaltic contractions and spike potentials in the stomach and small intestine of dogs [25]. Peripheral OT administration (120–240 µg/kg, i.p.) was shown to reduce gastric emptying and intestinal transit [21,22]. Similarly, subcutaneous OT (100 µg/kg) delayed gastric emptying and intestinal transit in mice [23]. Furthermore, microinjection of OT into the dorsal vagal complex (0.15, 0.2, and 0.3 µg/rat) led to a dose-dependent decrease in gastric tone and contractions [26]. In rats, both intracerebroventricular (i.c.v.) OT administration (10 µg/rat) and electrical stimulation of the paraventricular nuclei (PVN) inhibited gastric motility, whereas PVN lesions enhanced this effect, suggesting the presence of a tonic inhibitory control mediated by oxytocinergic pathways [13,27]. Consistent with these findings, our results further support the inhibitory role of OT (4–32 µg/kg, i.p.) in gastrointestinal motility. Notably, our study provides the first direct evidence that this neuropeptide suppresses jejunal MMC activity during fasting. Since MMC constitutes the physiological basis of interdigestive motility, these findings indicate that OT modulates not only postprandial motor functions (e.g., gastric emptying and intestinal transit) but also fasting-associated intestinal activity. Importantly, this inhibitory effect was evident even at intraperitoneal doses lower than those used in most previous studies, which primarily focused on postprandial conditions.

Despite the predominant evidence for inhibitory actions, several studies have also reported excitatory effects of OT on gastrointestinal motility under certain conditions. Both experimental animal studies and clinical studies have documented such stimulatory responses. In rabbits, i.v. (0.2–0.8 µg/kg) OT increased gastric and small intestinal pressure, indicating enhanced motility [28]. Similarly, in experimental animal models, centrally administered OT (0.1 and 0.5 µg, i.c.v.) normalized stress-induced delays in gastric emptying and restored gastric motility [29], while chronic peripheral pretreatment with OT (100 µg/kg/day, i.p., for 14 days) ameliorated vincristine-induced inhibition of intestinal motility [30]. In clinical studies, i.v. infusion of OT at a dose of 0.3 µg/kg has been shown to accelerate gastric emptying in humans [12], while lower-dose intravenous infusions administered at fixed rates (20–40 mU/min) increased colonic motility in healthy women [15]. Moreover, intranasal OT doubled defecation frequency in women with chronic constipation [31]. According to the current literature, the effects of OT on gastrointestinal motility are not unidirectional. These effects vary depending on multiple factors, including species, sex, administered dose (supraphysiological or physiological), route of administration, gastrointestinal segment, and physiological conditions. While OT generally exhibits stimulatory effects in humans, inhibitory responses appear to be more predominant in animals. Therefore, the role of OT in gastrointestinal motility should be regarded as that of a multifactorially regulated modulator [12].

Our study clearly demonstrated that the inhibitory effect of OT is mediated via OTRs, as pretreatment with the selective OTRs antagonist atosiban completely abolished this effect. This finding indicates that the action of OT on fasting intestinal motility is mediated by a receptor-specific mechanism, in alignment with previous reports in the literature. Indeed, Wu et al. reported that the inhibitory effects of i.p. OT on gastric emptying and intestinal transit in rats were mediated through OTRs [20,21]. Similarly, Flanagan and colleagues showed that i.c.v. OT suppressed gastric motility via OTR-dependent mechanisms [13,27]. In another study, Rogers and Hermann (1987) demonstrated that direct microinjection of OT into the dorsal vagal nucleus (DVC) reduced gastric motility, and this effect was prevented by co-administration of an OTR antagonist [32].

These results collectively suggest that the inhibitory effect of OT on MMC activity may arise from the activation of OTRs at both central and peripheral levels [12]. At the central level, activation of OTRs within the DVC has been shown to attenuate vagal efferent output, thereby suppressing gastrointestinal motor activity [22,32,33]. In contrast, OTRs located within the enteric nervous system and on smooth muscle cells may mediate direct peripheral effects [34,35]. While studies in humans indicate that OT-induced excitatory responses are mainly mediated by OTRs expressed on smooth muscle cells [8], findings from animal models suggest that the inhibitory actions of OT may involve central OTR-dependent pathways—such as those involving the DVC—or via indirect mechanisms, including the stimulation of endogenous cholecystokinin (CCK) release [12,20,21,33]. However, there is evidence indicating that endogenous CCK does not exert a modulatory effect on the small intestinal MMC pattern [36,37]. Accordingly, it appears unlikely that the inhibition of MMC activity following OT administration is mediated by a CCK-dependent mechanism. In our study, the OT-induced inhibition of MMC activity observed in fasted rats may involve OTR-dependent mechanisms at central and/or peripheral sites.

Previous studies have demonstrated that GLP-1 inhibits the MMC via activation of GLP-1Rs [16,17]. Accordingly, in the present study, we evaluated whether the inhibitory effect of OT on the MMC is related to GLP-1–GLP-1R signaling. The partial attenuation of OT’s inhibitory effect by a GLP-1R antagonist suggests that this signaling pathway contributes to OT-induced suppression of MMC activity. Although endogenous GLP-1 release and the activation of specific GLP-1-dependent neural circuits were not directly assessed in the present study, several observations from previous studies provide plausible mechanistic explanations for this interaction. Preproglucagon (PPG) neurons located in the nucleus tractus solitarii (NTS) have been shown to express OTRs and produce GLP-1 [38]. Moreover, exogenous OT has been reported to activate these OTRs on PPG neurons, thereby enhancing GLP-1 release and eliciting anorexigenic responses [38,39]. Consistent with our findings, PPG neurons project to the dorsal motor nucleus of the vagus (DMV), a key autonomic center regulating gastrointestinal motility. Importantly, direct GLP-1 administration into the DMV has been shown to inhibit gut motor activity in vivo [40,41], suggesting that OT–GLP-1 signaling may contribute to the MMC suppression observed in our study. While additional OTR-mediated mechanisms are likely involved, our findings align with recent evidence that OT also promotes GLP-1 secretion from pancreatic α-cells, further supporting a functional interaction between OT and GLP-1 systems [42].

In contrast, nitric oxide does not appear to mediate the inhibitory effect of OT on jejunal MMC activity. Although nitrergic signaling is known to tonically inhibit small intestinal motility and regulate MMC activity [43,44], inhibition of nitric oxide synthase did not alter the effect of OT in the present study. Consistent with earlier reports, OT appears to exert NO-dependent inhibitory effects predominantly in the colon, with no such activity detected in the jejunum [45,46].

The current study has several limitations that should be acknowledged. The present findings indicate that the suppressive effect of OT on jejunal MMC may involve central and/or peripheral mechanisms; however, the contribution of specific brainstem nuclei or defined neural pathways could not be directly assessed, as no targeted central interventions were performed in this study. Moreover, the relative contributions of central and peripheral OTR activation could not be clearly distinguished under the current experimental conditions. Further investigations using approaches such as selective vagotomy or region-specific pharmacological manipulations within the brainstem will be required to delineate the neural circuits mediating the effects of OT on fasting intestinal motility.

## 5. Conclusions

In conclusion, the present study provides direct experimental evidence that OT exerts a dose-dependent inhibitory effect on jejunal MMC activity in fasted, conscious male rats. This inhibitory response was fully abolished by pretreatment with the selective OTR antagonist atosiban, confirming the involvement of OTR-mediated mechanisms. Notably, the partial attenuation of this effect by a GLP-1 receptor antagonist suggests that OT–GLP-1R signaling may contribute to the suppression of MMC activity. In contrast, inhibition of nitric oxide synthase did not alter OT’s action, consistent with previous reports indicating that nitrergic pathways are not involved in jejunal OT responses. Collectively, these findings indicate that OT modulates fasting-associated small intestinal motility through OTR-dependent mechanisms, with partial involvement of GLP-1R signaling. Future studies employing region-specific pharmacological interventions or targeted neural manipulations will be necessary to further elucidate the precise neuroanatomical pathways underlying these effects.

## Data Availability

The data presented in this study are available from the corresponding author upon reasonable request.

## Abbreviations

The following abbreviations are used in this manuscript:

CCK	Cholecystokinin
DMV	Dorsal Motor Nucleus of the Vagus
DVC	Dorsal Vagal Complex
EMG	Electromyography
GLP-1	Glucagon-Like Peptide-1
GLP-1R	Glucagon-Like Peptide-1 Receptor
I.c.v.	Intracerebroventricular
I.p	Intraperitoneal
I.v.	Intravenous
L-NNA	NG-nitro-L-arginine
MMC	Migrating Myoelectric Complex
NO	Nitric Oxide
NOS	Nitric Oxide Synthase
NTS	Nucleus Tractus Solitarii
OT	Oxytocin
OTR	Oxytocin Receptor
PPG	Preproglucagon
